# Supplementary material for: Mechanically activated snai1b coordinates the initiation of myocardial delamination for trabeculation
Source: Nat Commun. 2025 Sep 24;16:8363. doi: 10.1038/s41467-025-62285-w (PMC12460811; doi:10.1038/s41467-025-62285-w)
Supplement: Supplementary file 1 — Supplementary Information [file 41467_2025_62285_MOESM1_ESM.pdf]

Mechanically Activated *snai1b* Coordinates the Initiation of Myocardial Delamination for Trabeculation

Jing Wang[1], Aaron L. Brown[2], Seul-Ki Park[3,4], Charlie Z. Zheng[5], Adam Langenbacher[5], Enbo Zhu[3,4], Ryan O'Donnell[3], Peng Zhao[3,4], Jeffrey J. Hsu[3], Tomohiro Yokota[3,4], Jiandong Liu[6], Jau-Nian Chen[5], Alison L. Marsden[7], Tzung K. Hsiai[1,3,4,*]

[1]Department of Bioengineering, University of California, Los Angeles, Los Angeles, CA, USA
[2]Department of Mechanical Engineering, Stanford University, Stanford, CA, USA
[3]Division of Cardiology, Department of Medicine, School of Medicine, University of California, Los Angeles, Los Angeles, CA, USA
[4]Department of Medicine, Greater Los Angeles Veteran Affairs Healthcare System, Los Angeles, CA, USA
[5]Department of Molecular, Cell, and Developmental Biology, University of California, Los Angeles, Los Angeles, CA, USA
[6]Department of Pathology and Laboratory Medicine, McAllister Heart Institute, University of North Carolina at Chapel Hill, NC, USA
[7]Departments of Pediatrics and Bioengineering, Stanford University, Stanford, CA, USA
*Correspondence: thsiai@mednet.ucla.edu

Supplementary Figures and Tables

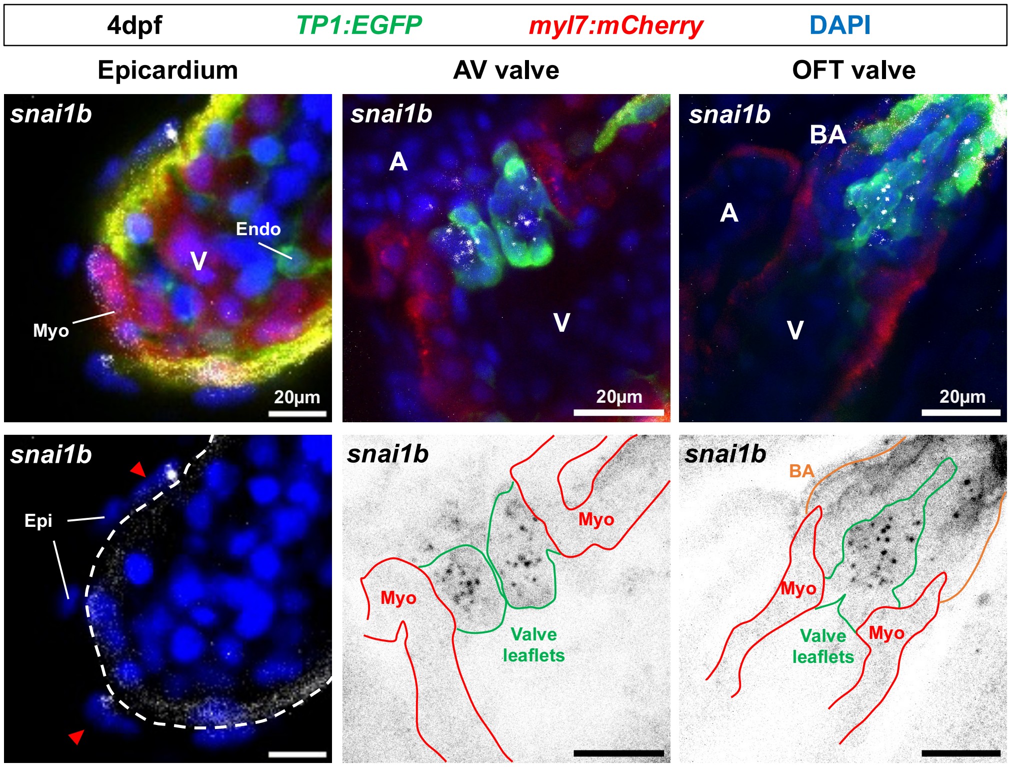

Supplementary Figure 1. Whole-mount *in situ* hybridization of *snai1b* mRNA in developing zebrafish hearts. Related to Figure 1.

snai1b is expressed in the epicardial (arrowheads) and valvular cells of developing zebrafish hearts at 4 dpf. The border of myocardium and valve leaflets is indicated by the Notch reporter *Tg(TP1: EGFP)*.

Anatomic labels: BA, bulbus arteriosus; V, ventricle; A, atrium; Myo, myocardium; Endo, endocardium; Epi, epicardium.

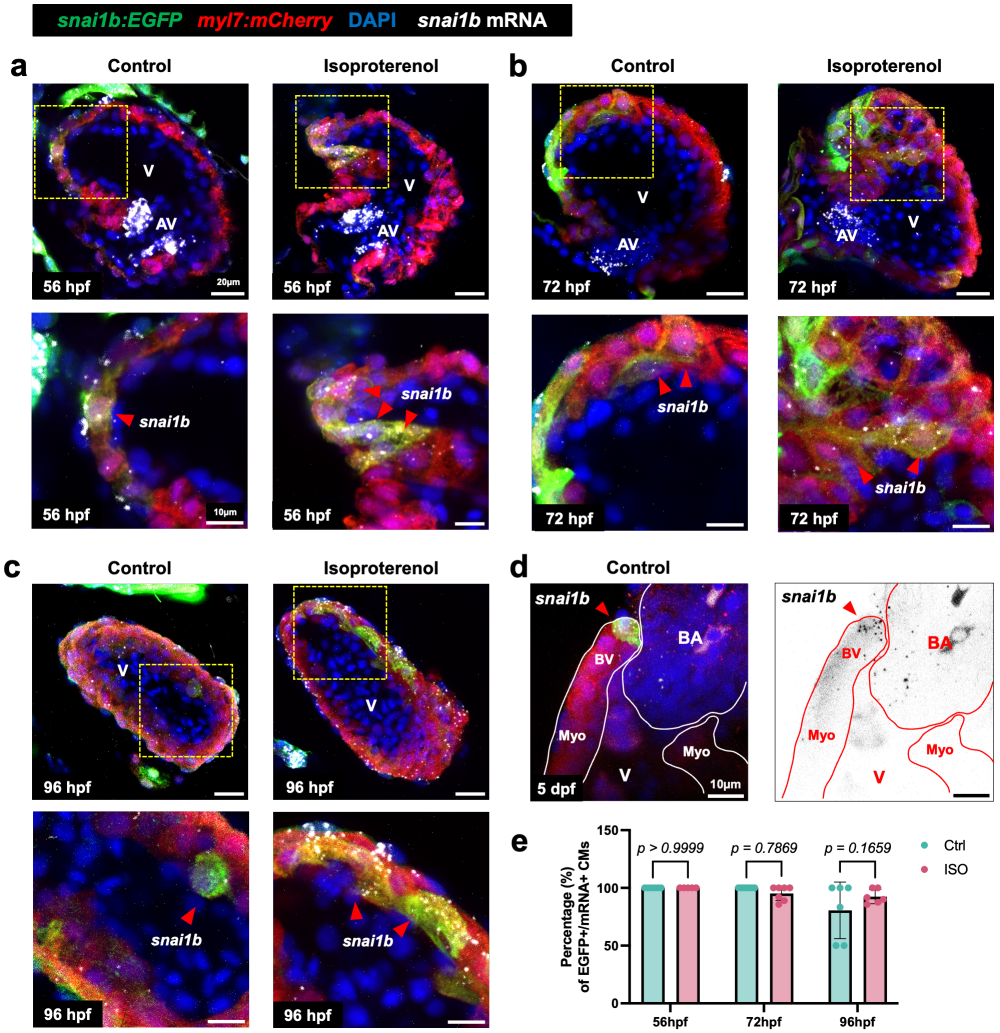

Supplementary Figure 2. Whole-mount *in situ* hybridization of *snai1b* mRNA in the larval hearts of *Tg(snai1b:EGFP; myl7:mCherry)* reporter line. Related to Figure 1.

(a-d) Whole-mount *in situ* hybridization of *snai1b* mRNA (arrowheads) reveals that the reporter activity is myocardial-specific at 56 hpf **(a)**, 72 hpf **(b)**, 96 hpf **(c)**, and 5 dpf **(d)**.

(e) Percentage of *snai1b:EGFP*-positive cardiomyocytes (CMs) that are also positive for mRNA staining at 56 hpf (Ctrl n = 6, ISO n = 5), 72 hpf (Ctrl n = 7, ISO n = 7), and 96 hpf (Ctrl n = 6, ISO n = 6). All values are displayed with mean and standard deviation (SD). *p*-value is displayed for each comparison. Ordinary two-way ANOVA followed by Sidak's multiple comparisons test on the means was applied to determine statistical significance. Source data are provided as a Source Data file.

Anatomic labels: BA, bulbus arteriosus; V, ventricle; AV, atrioventricular canal; BV, bulbus-ventricular annulus; Myo, myocardium.

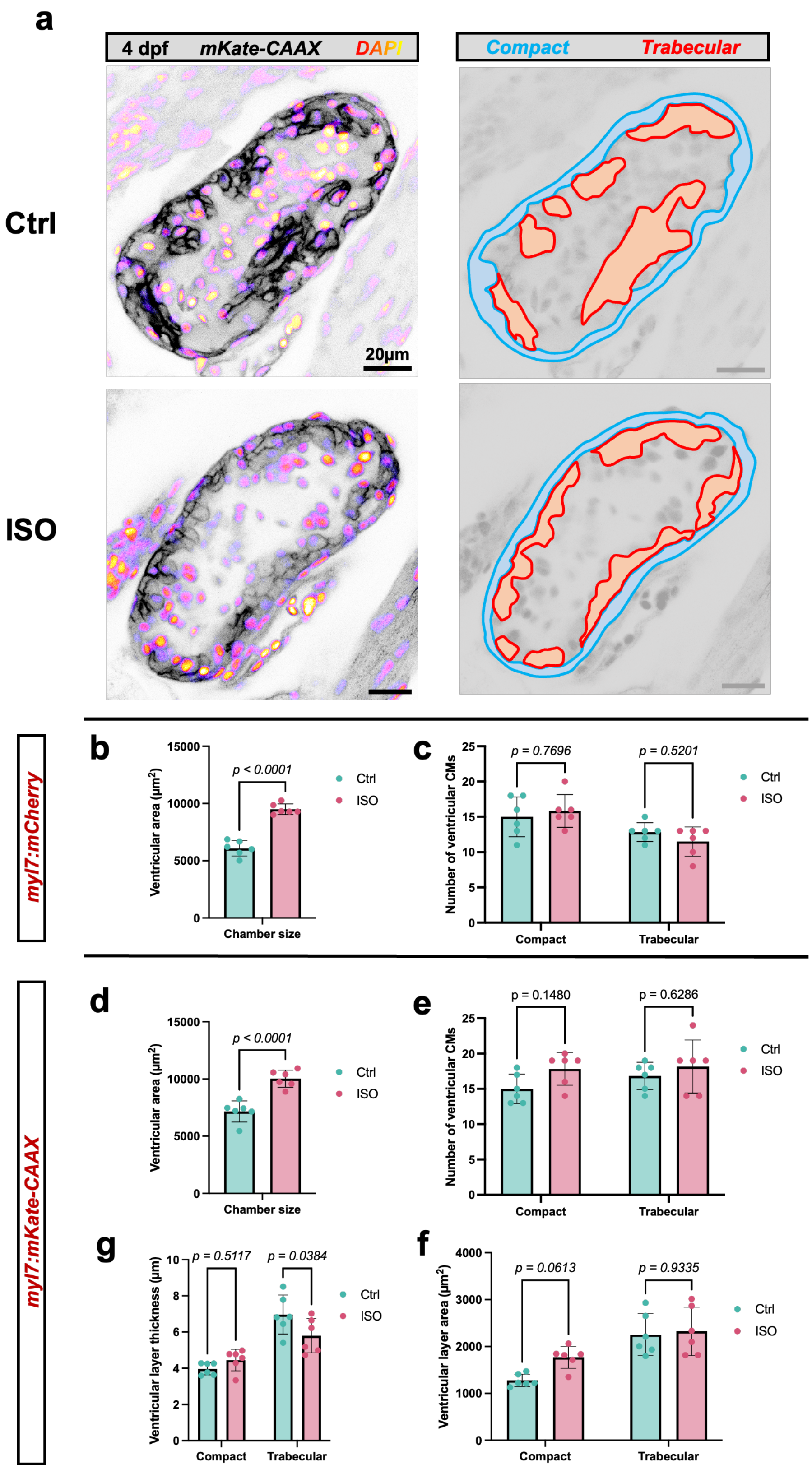
a
4 dpf mKate-CAAX DAPI
Compact Trabecular
Ctrl
ISO
20µm
myl7:mCherry
myl7:mKate-CAAX
b
Ventricular area (µm²)
p < 0.0001
Chamber size
Ctrl
ISO
c
Number of ventricular CMs
p = 0.7696
p = 0.5201
Compact
Trabecular
d
p < 0.0001
e
p = 0.1480
p = 0.6286
g
Ventricular layer thickness (µm)
p = 0.5117
p = 0.0384
f
Ventricular layer area (µm²)
p = 0.0613
p = 0.9335

Supplementary Figure 3. ISO-induced alteration in ventricular compact vs. trabecular morphology. Related to Figure 2.

(a) Representative images of control vs. ISO-treated *Tg(myl7:mKate-CAAX)* hearts at 4 dpf with nuclei labeled by DAPI. The right panels outline the morphology of compact and trabecular layers.

(b-e) ISO significantly increased the ventricle's cross-sectional area but not the number of either compact or trabecular CMs, as quantified using both *Tg(myl7:mCherry)* and *Tg(myl7:mKate-CAAX)* hearts.

(f-g) ISO did not change the absolute size of either the compact or trabecular layer but significantly reduced the thickness of the trabecular layer, while the thickness of the compact layer remained the same.

Six control and Six ISO-treated hearts were analyzed for each of the two reporter lines. All values are displayed with mean and standard deviation (SD). *p*-value is displayed for each comparison. Ordinary two-way ANOVA followed by Sidak's multiple comparisons test on the means was applied to determine statistical significance, except for panels b and d, which used paired t-test (two-tailed). Source data are provided as a Source Data file.

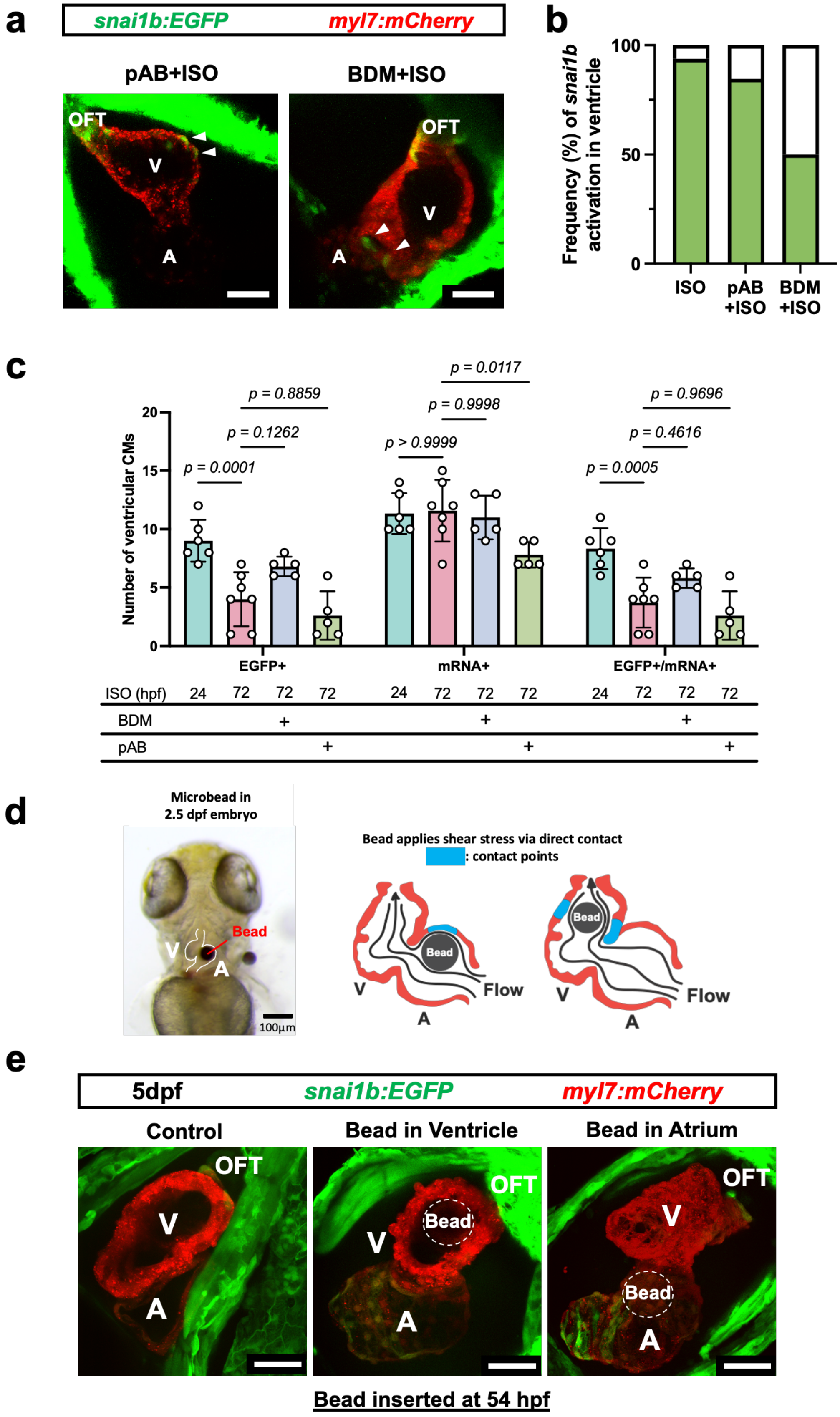

a
snai1b:EGFP
myl7:mCherry
pAB+ISO
BDM+ISO
OFT
V
A
b
Frequency (%) of snai1b activation in ventricle
100
50
0
ISO
pAB +ISO
BDM +ISO
c
Number of ventricular CMs
p = 0.0001
p = 0.1262
p = 0.8859
p > 0.9999
p = 0.9998
p = 0.0117
p = 0.0005
p = 0.4616
p = 0.9696
EGFP+
mRNA+
EGFP+/mRNA+
ISO (hpf)
24
72
BDM
pAB
d
Microbead in 2.5 dpf embryo
Bead
100µm
Bead applies shear stress via direct contact
: contact points
Flow
e
5dpf
Control
Bead in Ventricle
Bead in Atrium
Bead inserted at 54 hpf

Supplementary Figure 4. Myosin inhibitor vs. shear stress in myocardial *snai1b* activation. Related to Figure 2.

(a-b) Hearts co-treated with ISO and myosin inhibitors at 1 dpf revealed a lower frequency of *snai1b* activation (arrowheads) in the ventricle at 5 dpf. pAB (n = 13): para-amino-blebbistatin; BDM (n = 6): 2,3-butanedione 2-monoxime. Source data are provided as a Source Data file.

(c) ISO treatment starting at 72 hpf instead of 24 hpf reveals a statistically significant decrease in $EGFP^+$ CMs, but not $mRNA^+$ CMs, using the whole-mount *in situ* hybridization of *snai1b* mRNA in *Tg(snai1b:EGFP; myl7:mCherry)* hearts at 96 hpf. Applying pAB along with ISO at 72 hpf led to a significant decrease of $mRNA^+$ CMs, compared to ISO alone. All values are displayed with mean and standard deviation (SD). *p*-value is displayed for each comparison. Number of hearts analyzed: ISO-24hpf = 6, ISO-72hpf = 7, ISO-BDM = 5, ISO-pAB = 5. Ordinary two-way ANOVA followed by Sidak's multiple comparisons test on the means was applied to determine statistical significance. Source data are provided as a Source Data file.

(d) Left panel highlights a microbead in the embryo's heart at 2.5 dpf. Right panel illustrates the concept of applying shear stress directly on the endocardium by the microbead.

(e) Microbead-inserted hearts exhibited no activation of *snai1b* in the ventricle at 5 dpf, regardless of the bead's location (sham n = 6, bead-inserted n = 9).

Anatomic labels: V, ventricle; A, atrium; OFT, outflow tract. Scale bars = 50 μm unless specified.

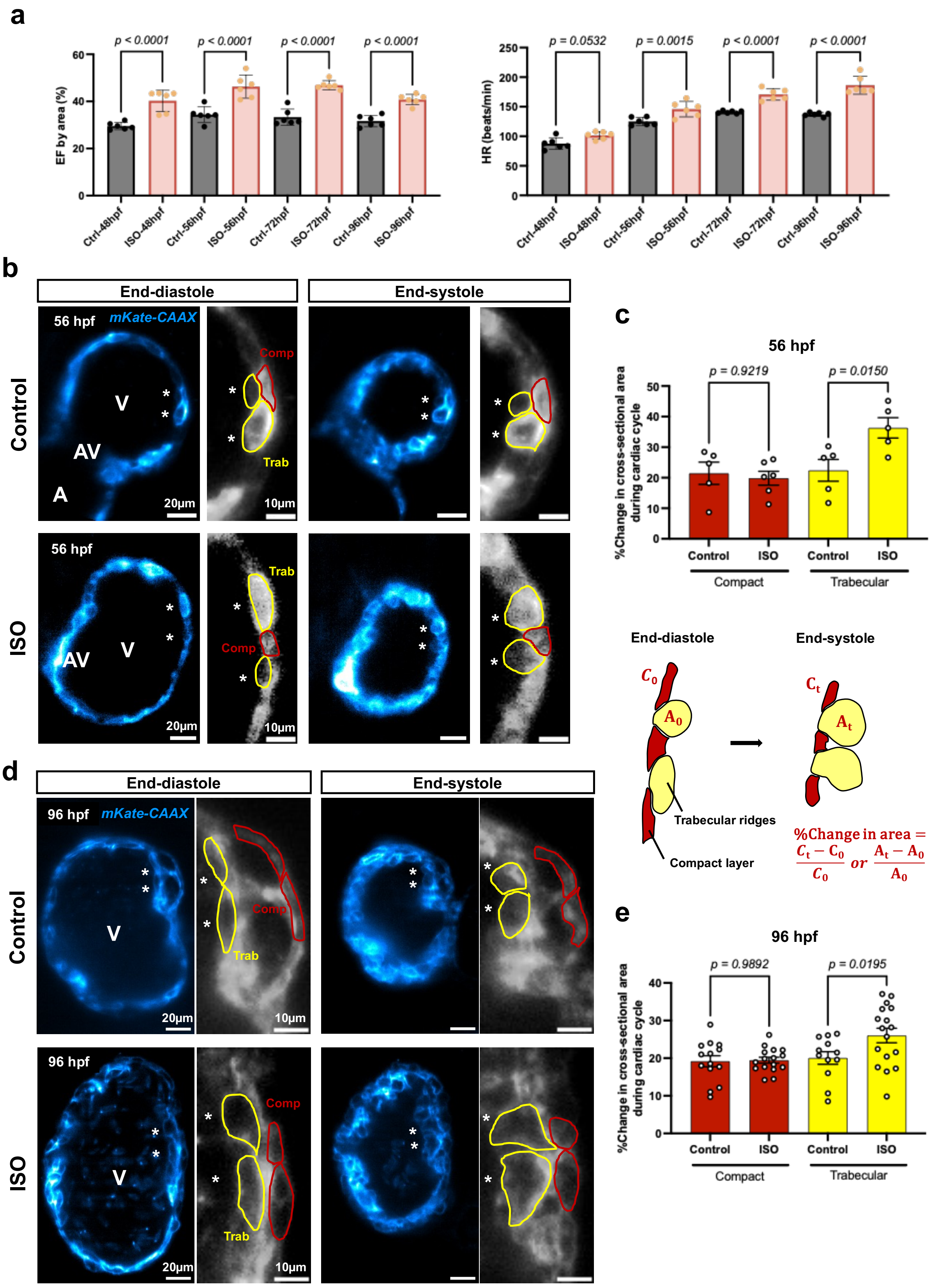

a
p < 0.0001
EF by area (%)
Ctrl-48hpf
ISO-48hpf
Ctrl-56hpf
ISO-56hpf
Ctrl-72hpf
ISO-72hpf
Ctrl-96hpf
ISO-96hpf
p = 0.0532
p = 0.0015
HR (beats/min)
b
End-diastole
End-systole
56 hpf
mKate-CAAX
Control
ISO
V
AV
A
Comp
Trab
20µm
10µm
c
56 hpf
%Change in cross-sectional area during cardiac cycle
p = 0.9219
p = 0.0150
Control
ISO
Compact
Trabecular
Trabecular ridges
Compact layer
%Change in area = $\frac{C_t - C_0}{C_0}$ or $\frac{A_t - A_0}{A_0}$
d
96 hpf
e
p = 0.9892
p = 0.0195

Supplementary Figure 5. ISO-mediated increase in the change of trabecular cross-sectional area during contraction. Related to Figure 3.

(a) ISO treatment induced a significant increase in ejection fraction (EF) and heart rate (HR) from 48 hpf to 96 hpf. All values are displayed with mean and standard deviation (SD). *p*-value is displayed for each comparison.

(b-e) 2-D cross-sections of the trabeculae (asterisks) reveal that the cross-sectional area of both compact (red dashed outlines) and trabecular (yellow dashed outlines) CMs enlarges during systole. At 56 hpf **(c)** and 96 hpf **(e)**, ISO treatment significantly increased the enlargement of the area within trabecular CMs, whereas the enlargement in the compact CMs remained similar. All values are displayed with mean and standard error of mean (SEM). *p*-value is displayed for each comparison.

Anatomic labels: V, ventricle; A, atrium; AV, AV canal. One control heart and one ISO-treated heart were used for analysis at 56 hpf. Two control hearts and two ISO-treated hearts were used for analysis at 96 hpf. Ordinary one-way ANOVA followed by Sidak's multiple comparisons test on the means was applied to determine statistical significance. Source data are provided as a Source Data file.

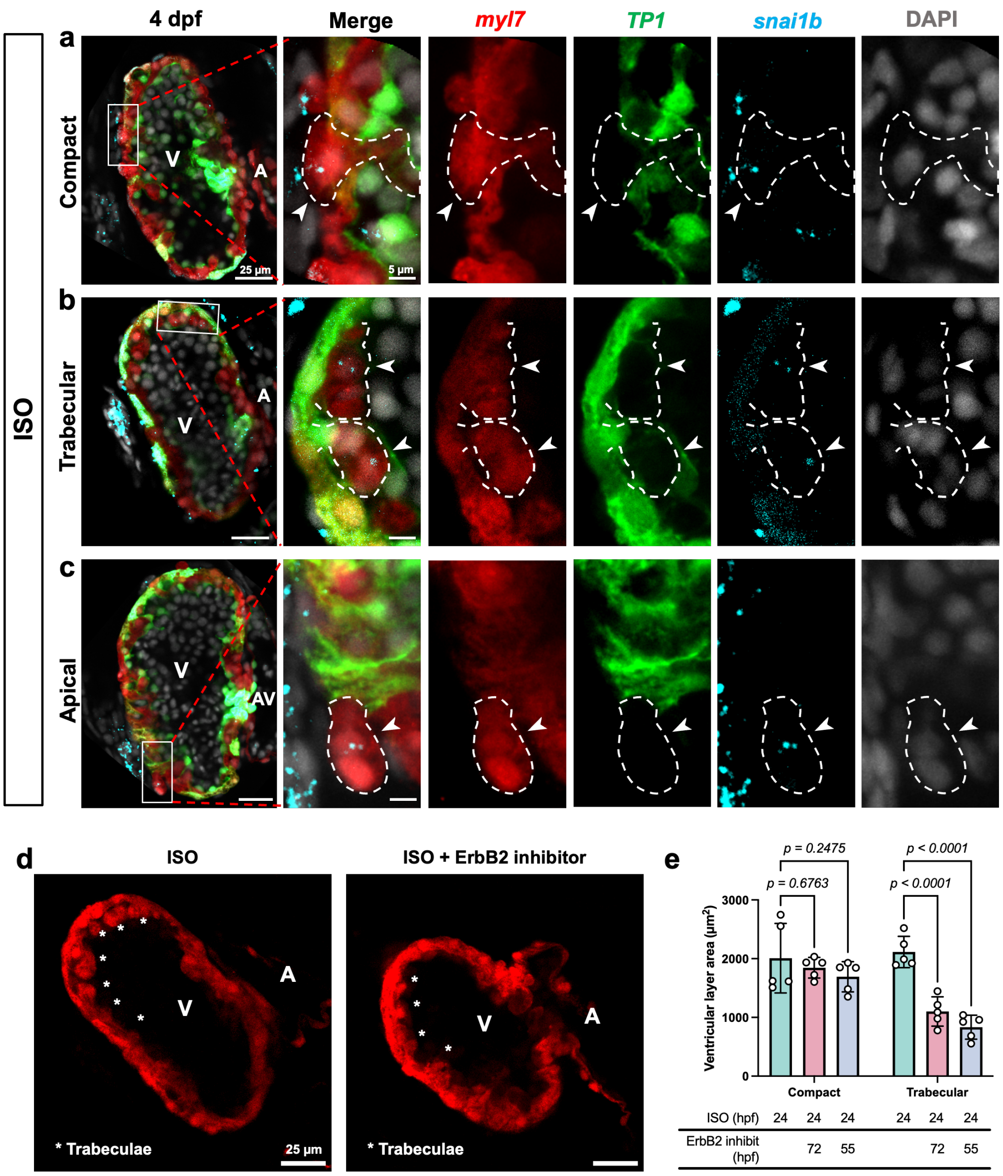

Supplementary Figure 6. ISO-induced *snai1b* activation in Notch-negative ($^-$) cardiomyocytes at 4 dpf. Related to Figure 4.

(a-c) ISO-induced *snai1b* (arrowheads) were observed in compact **(a)**, trabecular **(b)**, and apically delaminated **(c)** Notch$^-$ cardiomyocytes (dashed outlines in magnified panels).

(d-e) Treating with ErbB2 inhibitor, PD168393, from 55 hpf or 72hpf significantly reduced the area of trabeculae at 4 dpf. 5 hearts were analyzed for each condition. Number of hearts analyzed: control n = 6; ISO n = 7; ISO+PD@72hpf n = 7; ISO+PD@55hpf n = 5. All values are displayed with mean and standard deviation (SD). *p*-value is displayed for each comparison. Ordinary two-

way ANOVA followed by Sidak's multiple comparisons test on the means was applied to determine statistical significance Source data are provided as a Source Data file.

Anatomic labels: V, ventricle; A, atrium; AV, atrioventricular canal.

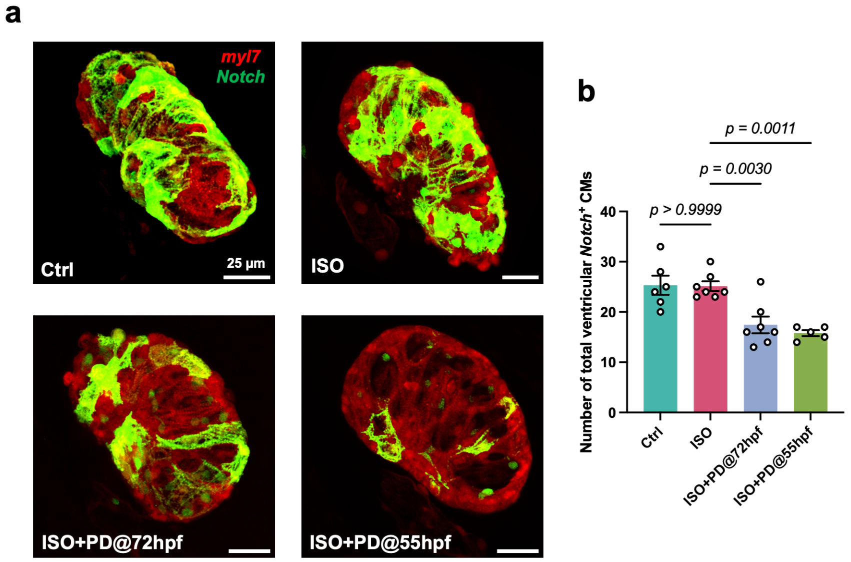

Supplementary Figure 7. Myocardial Notch activity in control vs. ISO-treated hearts. Related to Figure 4.

(a) Representative images of myocardial Notch activity in control, ISO-treated, and ISO-ErbB2 inhibitor-cotreated hearts at 96 hpf.

(b) ErbB2 inhibitor (PD) significantly reduced the total number of $Notch^{+}$ CMs per ventricle, while ISO alone did not. Number of hearts analyzed: control n = 6; ISO n = 7; ISO+PD@72hpf n = 7; ISO+PD@55hpf n = 5. All values are displayed with mean and standard error of mean (SEM). *p*-value is displayed for each comparison. Ordinary one-way ANOVA followed by Sidak's multiple comparisons test on the means was applied to determine statistical significance. Source data are provided as a Source Data file.

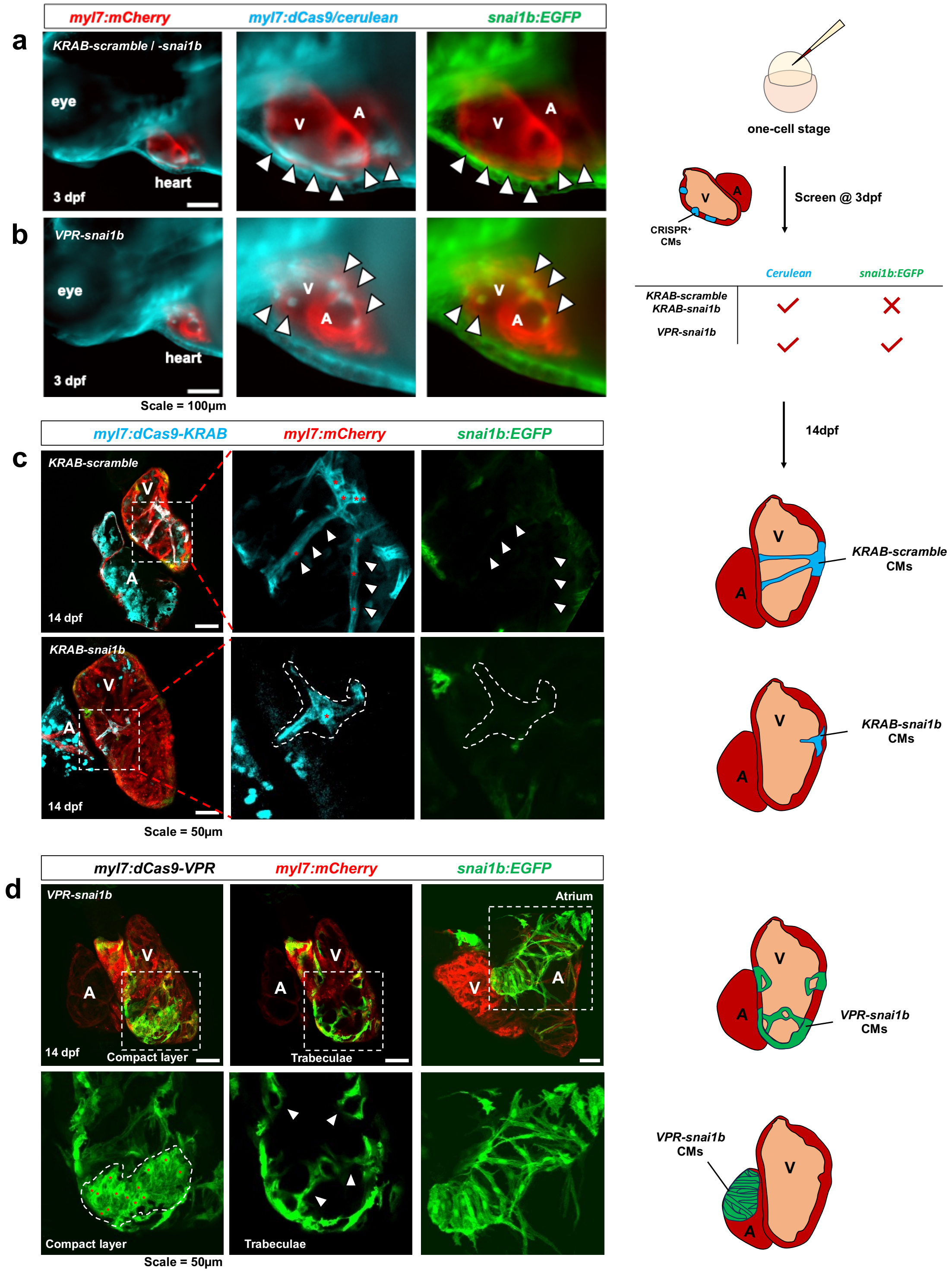

myl7:mCherry
myl7:dCas9/cerulean
snai1b:EGFP
a
KRAB-scramble / -snai1b
eye
heart
3 dpf
V
A
b
VPR-snai1b
eye
heart
3 dpf
V
A
Scale = 100µm
one-cell stage
V
A
CRISPR+ CMs
Screen @ 3dpf
Cerulean
snai1b:EGFP
KRAB-scramble
KRAB-snai1b
VPR-snai1b
14dpf
c
myl7:dCas9-KRAB
myl7:mCherry
snai1b:EGFP
KRAB-scramble
V
A
14 dpf
KRAB-snai1b
V
A
14 dpf
Scale = 50µm
KRAB-scramble CMs
KRAB-snai1b CMs
d
myl7:dCas9-VPR
myl7:mCherry
snai1b:EGFP
VPR-snai1b
V
A
14 dpf
Compact layer
Trabeculae
Atrium
Compact layer
Trabeculae
Scale = 50µm
VPR-snai1b CMs
VPR-snai1b CMs

Supplementary Figure S8. Validation of myocardial Tol2-CRISPR interference system. Related to Figure 5.

(a-b) At 3 dpf, cerulean-positive ($^+$) cardiomyocytes (CMs, arrowheads) were found in hearts after the injection of dCas9-KRAB with scramble or *snai1b*-targeting sgRNAs. In both groups, there is no expression of *snai1b*, while cerulean$^+$ CMs injected with dCas9-VPR expressed *snai1b*.

(c-d) At 14 dpf, the Tol2-CRISPR system continued to express in scramble (n = 7), KRAB-*snai1b* (repression, n = 8), and VPR-*snai1b* (activation, n = 9) CMs. Control CMs formed long trabecular fibers (arrowheads, nuclei - asterisks), whereas *snai1b*-repressed CMs constricted to small colonies (dashed outlines). *snai1b*-activated CMs developed in large areas of the compact layer (dashed outlines) and the trabeculation (arrowheads).

Anatomic labels: V, ventricle; A, atrium.

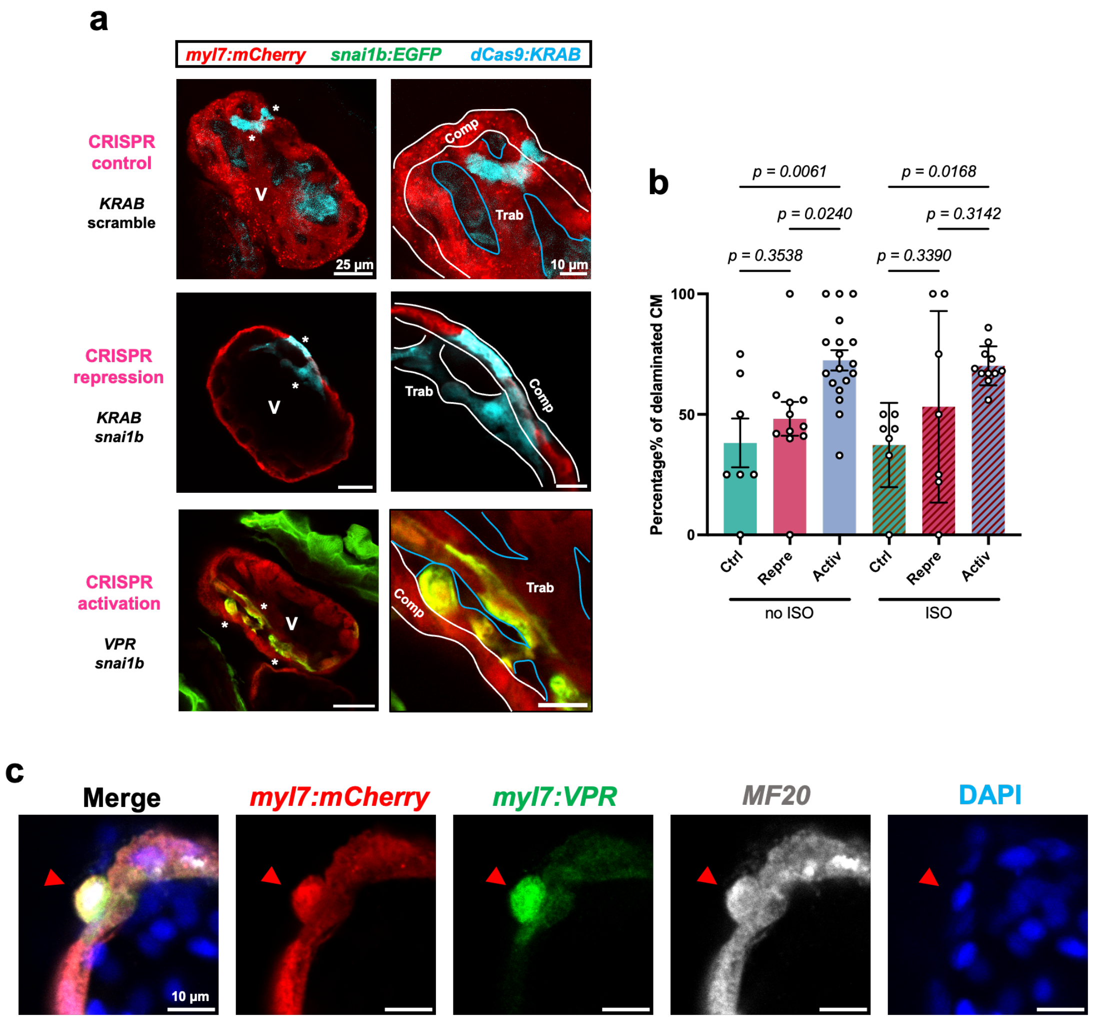

Supplementary Figure 9. Activation and repression of *snai1b* to modulate delamination for trabeculation (no ISO). Related to Figure 5.

(a) Representative images of CRISPRa/i-injected hearts at 4 dpf (96 hpf) without ISO.

(b) Percentage of delaminated (trabecular and apical) CMs in each ventricle across conditions. ISO did not significantly alter the delamination of CMs within the same CRISPRa/i groups. However, *snai1b*-repression under ISO treatment led to 37.5% of CMs undergoing apical delamination and 6.7% trabeculation, compared to 41.7% undergoing trabeculation in repression-only hearts (Figure 5d). All values are displayed with mean and standard error of mean (SEM). *p*-value is displayed for each comparison. Number of hearts analyzed: Control = 7, Repression = 11, Activation = 18. Ordinary one-way ANOVA followed by Holm-Sidak's multiple comparisons test on the means was applied to determine statistical significance. Source data are provided as a Source Data file.

(c) Staining of a myocardial-specific marker, myosin 4 (MF20), in apically delaminated CMs (arrowhead).

Anatomic labels: V, ventricle; Comp, compact layer; Trab, trabeculae.

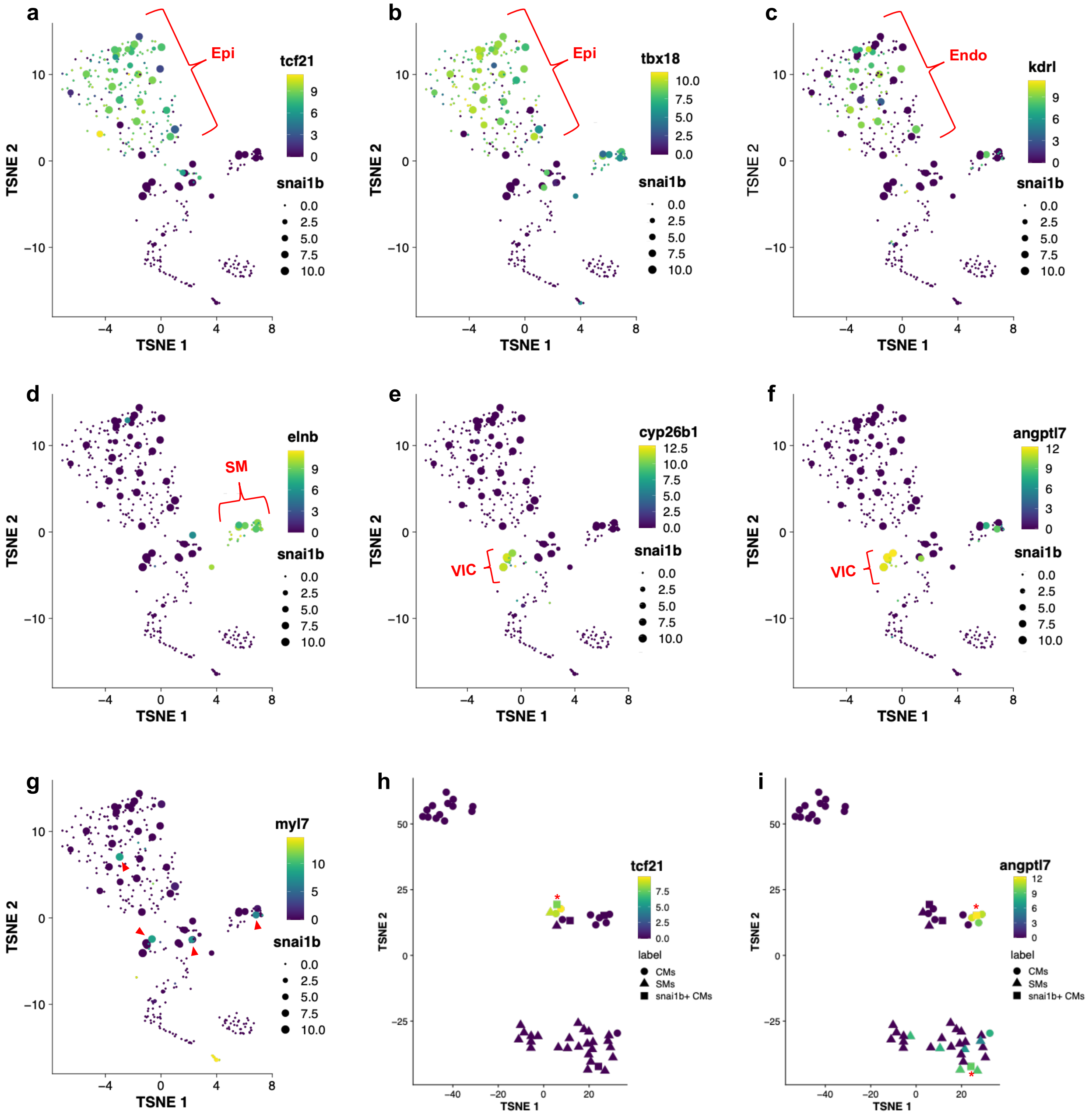

Supplementary Figure 10. ***snai1b*****-expressing CMs among the sequenced cells at 5 dpf. Related to Figure 6.**

(a-c) The largest clusters of *snai1b*$^+$ cells came from the epicardial (Epi, *tcf21*$^+$/*tbx18*$^+$) and the endocardial (Endo, *kdrl*$^+$) cells. The dot size indicates the expression level (normalized log counts) of *snai1b*. The color scale depicts that of marker genes.

(d-f) *snai1b* was also expressed in the bulbus SMs (*elnb*$^+$) and the valvular interstitial cells (VIC, *cyp26b1*$^+$/*angptl7*$^+$).

(g-i) The dataset solely contained 4 *snai1b*$^+$ CMs (arrowheads), where 3 also expressed epicardial (*tcf21*) or VIC (*angptl7*) markers (asterisks).

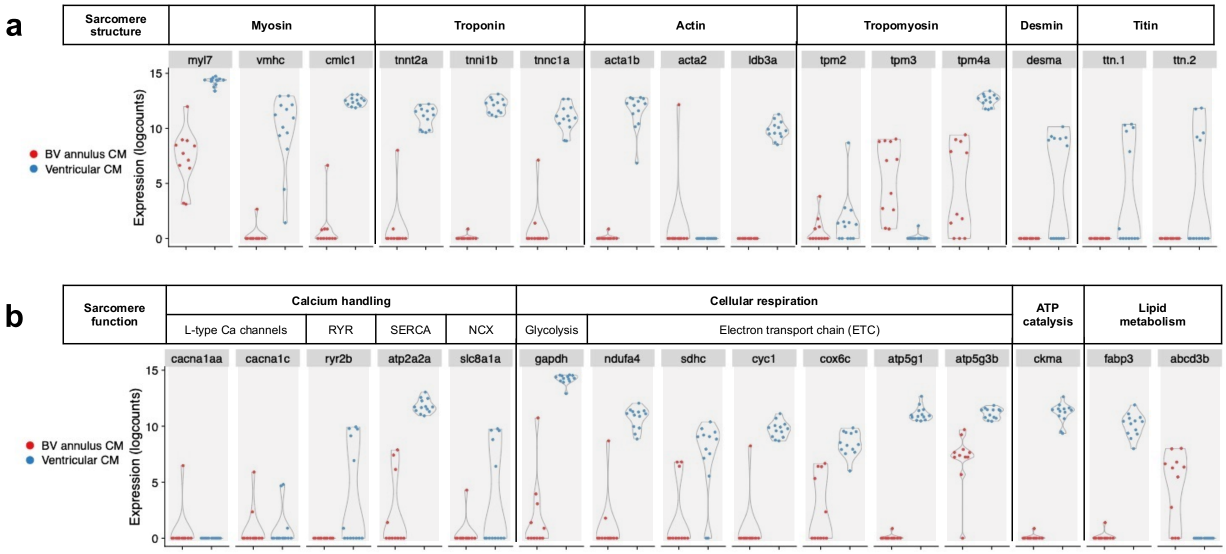

Supplementary Figure 11. Lower expression of sarcomeric genes in BV annulus CMs than in ventricular CMs. Related to Figure 6.

(a-b) Genes related to sarcomeric structure, calcium handling, and electron transport machinery were downregulated in BV annulus CMs, indicating that their actomyosin network shifted to a mesenchymal-like state.

Supplementary Table 1

REAGENT or RESOURCE	SOURCE	IDENTIFIER
Antibodies		
Rabbit anti-GFP polyclonal	GeneTex	GTX113617
Rat anti-mCherry monoclonal	Invitrogen	M11217
Mouse MF20 monoclonal	Invitrogen	14-6503-82
Goat anti-Rabbit IgG (H+L), Alexa Fluor 488	Invitrogen	A-11008
Goat anti-Rat IgG (H+L), Alexa Fluor 594	Invitrogen	A-11007
Goat anti-Mouse IgG (H+L), Alexa Fluor 647	Invitrogen	A-21235
Chemicals, Peptides, and Recombinant Proteins		
1-phenyl-2-thiourea (PTU)	Sigma-Aldrich	P7629
Tricaine/MS-222	Sigma-Aldrich	E10521
TopVision Low Melting Point Agarose	Thermo Fisher	R0801
DAPI	Thermo Fisher	D1306
Isoproterenol hydrochloride (ISO)	Sigma-Aldrich	I5627
2,3-butanedione 2-monoxime (BDM)	Sigma-Aldrich	B0753
para-amino-blebbistatin (pAB)	Cayman Chemical	22699
PD168393	Cayman Chemical	21059
LY364947	Abcam	ab141890
magnetic silica microbeads	Alpha Nanotech	50 µm
Critical Commercial Assays		
RNAscope™ Multiplex Fluorescent Detection Kit v2	Advanced Cell Diagnostics	323110
RNAscope Probe-Dr-snai1b	Advanced Cell Diagnostics	505101-C3
RNAscope Probe-Dr-col1a2	Advanced Cell Diagnostics	526061-C2
RNAscope 3-plex Negative Control Probe (DapB)	Advanced Cell Diagnostics	320871
Opal 520	Akoya Biosciences	NC1601877
Opal 690	Akoya Biosciences	NC1605064
Co-Detection Antibody Diluent	Advanced Cell Diagnostics	323160
Experimental Models: Organisms/Strains		
Tg(myl7:mCherry)	UCLA fish core and Hsiai Lab	N/A
Tg(flk:mCherry)	UCLA fish core and Hsiai Lab	N/A
Tg(snai1b:EGFP)	Stewart Lab and Hsiai Lab	Jimenez *et al.*, Disease Models & Mechanisms (2016)
Tg(*myl7:mCherry-zCdt1*)	Poss Lab and Hsiai Lab	Choi *et al.*, Development (2013)

Tg(TP1:EGFP)	Traver Lab, Lawson Lab, and Hsiai Lab	*Parsons et al.*, Mechanisms of Development (2009)
Oligonucleotides		
Tol2 transposase mRNA	VectorBuilder	R008S
Recombinant DNA		
myl7(cmlc2)-dCas9-KRAB-cerulean plasmid	VectorBuilder	VB230511-1580rdq
myl7(cmlc2)-dCas9-VPR-cerulean plasmid	VectorBuilder	VB230511-1582zcv
myl7(cmlc2)-dCas9-KRAB-EGFP plasmid	VectorBuilder	VB240619-1578kbk
myl7(cmlc2)-dCas9-VPR-EGFP plasmid	VectorBuilder	VB240619-1579byz
U6-sgRNA#1-2 plasmid	VectorBuilder	VB230805-1121bhp
U6-sgRNA#3-4 plasmid	VectorBuilder	VB230805-1122cpk
U6-scramble#1-2 plasmid	VectorBuilder	VB231031-1011szs
Software and Algorithms		
FIJI v2.9.0	Schindelin *et al.*, Nature Methods (2012)	RRID:SCR_002285
LAS X	Leica	3.5.7
Micro-Manager	Edelstein *et al.*, Journal of Biological Methods (2014)	1.4 & 2.0
MATLAB R2019a	MathWorks	RRID:SCR_001622
GraphPad Prism v9.5.0	GraphPad Software	RRID:SCR_002798
ParaView v5.11.0	Kitware	RRID:SCR_002516
SimpleITK v2.2.1	Lokekamp *et al.*, Frontiers in Neuroinformatics (2013)	RRID:SCR_024693
scikit-image v19.3	van der Walt *et al.*, PeerJ (2014)	RRID:SCR_021142
3D Slicer v5.2.1	Fedorov *et al.*, Magnetic Resonance Imaging (2012)	RRID:SCR_005619
Amira	Thermo Fisher	6.0.1
R v4.1.2	R Core Team	RRID:SCR_001905
RStudio v2023.03.0+386	Posit Software	RRID:SCR_000432
scater v1.22.0	McCarthy *et al.*, Bioinformatics (2017)	RRID:SCR_015954
scran v1.22.1	Bioconductor	RRID:SCR_016944
clusterProfiler v4.2.2	Wu *et al.*, The Innovation (2021)	RRID:SCR_016884
enrichplot v1.14.2	Bioconductor	N/A
Single-cell RNA seq analysis code	Zenodo	10.5281/zenodo.10525228
