# Supplementary material for: Mechanically activated snai1b coordinates the initiation of myocardial delamination for trabeculation
Source: Nat Commun. 2025 Sep 24;16:8363. doi: 10.1038/s41467-025-62285-w (PMC12460811; doi:10.1038/s41467-025-62285-w)
Supplement: Supplementary file 2 — Reporting Summary [file 41467_2025_62285_MOESM2_ESM.pdf]

nature portfolio

Corresponding author(s): Tzung K. Hsiai

Last updated by author(s): Jun 25, 2025

Reporting Summary

Nature Portfolio wishes to improve the reproducibility of the work that we publish. This form provides structure for consistency and transparency in reporting. For further information on Nature Portfolio policies, see our Editorial Policies and the Editorial Policy Checklist.

Statistics

For all statistical analyses, confirm that the following items are present in the figure legend, table legend, main text, or Methods section.

n/a	Confirmed	
☐	☒	The exact sample size (*n*) for each experimental group/condition, given as a discrete number and unit of measurement
☐	☒	A statement on whether measurements were taken from distinct samples or whether the same sample was measured repeatedly
☐	☒	The statistical test(s) used AND whether they are one- or two-sided<br>*Only common tests should be described solely by name; describe more complex techniques in the Methods section.*
☒	☐	A description of all covariates tested
☐	☒	A description of any assumptions or corrections, such as tests of normality and adjustment for multiple comparisons
☐	☒	A full description of the statistical parameters including central tendency (e.g. means) or other basic estimates (e.g. regression coefficient) AND variation (e.g. standard deviation) or associated estimates of uncertainty (e.g. confidence intervals)
☐	☒	For null hypothesis testing, the test statistic (e.g. *F*, *t*, *r*) with confidence intervals, effect sizes, degrees of freedom and *P* value noted<br>*Give P values as exact values whenever suitable.*
☒	☐	For Bayesian analysis, information on the choice of priors and Markov chain Monte Carlo settings
☒	☐	For hierarchical and complex designs, identification of the appropriate level for tests and full reporting of outcomes
☒	☐	Estimates of effect sizes (e.g. Cohen's *d*, Pearson's *r*), indicating how they were calculated

Our web collection on statistics for biologists contains articles on many of the points above.

Software and code

Policy information about availability of computer code

Data collection	For confocal and digital light-sheet imaging, we used LAS X (ver 3.5.7). For spinning disk confocal, we used Micro-Manager (ver 2.0) with custom control code. For epi-fluorescence imaging, we used Micro-Manager (ver 1.4).
Data analysis	All the analysis software used in this study can be found in Supplementary Table 1. The original R script for scRNA-seq analysis can be accessed via Zenodo repository [http://doi.org/10.5281/zenodo.10525228]. Its DOI is listed in Supplementary Table 1. The code for 4D imaging synchronization and myocardial strain mapping is custom-written and involve multiple software. Due to this non-turnkey nature, the code is not uploaded to public repository and can be shared by the lead contact with requests. The source and RRIDs of the analysis software used in this study can be found in Supplementary Table 1 (FIJI v2.9.0, MATLAB R2019a, GraphPad Prism v9.5.0, ParaView v5.11.0, SimpleITK v2.2.1, scikit-image v19.3, 3D Slicer v5.2.1, Amira v6.0.1, R v4.1.2, RStudio v2023.03.0+386, scater v1.22.0, scran v1.22.1, clusterProfiler v4.2.2, enrichplot v1.14.2).

For manuscripts utilizing custom algorithms or software that are central to the research but not yet described in published literature, software must be made available to editors and reviewers. We strongly encourage code deposition in a community repository (e.g. GitHub). See the Nature Portfolio guidelines for submitting code & software for further information.

Data

Policy information about availability of data

All manuscripts must include a data availability statement. This statement should provide the following information, where applicable:

- Accession codes, unique identifiers, or web links for publicly available datasets
- A description of any restrictions on data availability
- For clinical datasets or third party data, please ensure that the statement adheres to our policy

The single-cell RNA seq dataset analyzed in this study can be downloaded from NCBI GEO (accession number GSE121750), where the post-QC matrix (M0) was used. Source data of all graphs in the paper is provided in the Source Data file. The R data file for single-cell RNA seq analysis and the VTK files for strain mapping can be accessed via Zenodo repository [http://doi.org/10.5281/zenodo.10525228]. Its DOI is also listed in Supplementary Table 1. Any additional information required to reanalyze the data reported in this paper is available from the lead contact upon request.

Research involving human participants, their data, or biological material

Policy information about studies with human participants or human data. See also policy information about sex, gender (identity/presentation), and sexual orientation and race, ethnicity and racism.

Reporting on sex and gender	*Use the terms sex (biological attribute) and gender (shaped by social and cultural circumstances) carefully in order to avoid confusing both terms. Indicate if findings apply to only one sex or gender; describe whether sex and gender were considered in study design; whether sex and/or gender was determined based on self-reporting or assigned and methods used.*<br>*Provide in the source data disaggregated sex and gender data, where this information has been collected, and if consent has been obtained for sharing of individual-level data; provide overall numbers in this Reporting Summary. Please state if this information has not been collected.*<br>*Report sex- and gender-based analyses where performed, justify reasons for lack of sex- and gender-based analysis.*
Reporting on race, ethnicity, or other socially relevant groupings	*Please specify the socially constructed or socially relevant categorization variable(s) used in your manuscript and explain why they were used. Please note that such variables should not be used as proxies for other socially constructed/relevant variables (for example, race or ethnicity should not be used as a proxy for socioeconomic status).*<br>*Provide clear definitions of the relevant terms used, how they were provided (by the participants/respondents, the researchers, or third parties), and the method(s) used to classify people into the different categories (e.g. self-report, census or administrative data, social media data, etc.)*<br>*Please provide details about how you controlled for confounding variables in your analyses.*
Population characteristics	*Describe the covariate-relevant population characteristics of the human research participants (e.g. age, genotypic information, past and current diagnosis and treatment categories). If you filled out the behavioural & social sciences study design questions and have nothing to add here, write "See above."*
Recruitment	*Describe how participants were recruited. Outline any potential self-selection bias or other biases that may be present and how these are likely to impact results.*
Ethics oversight	*Identify the organization(s) that approved the study protocol.*

Note that full information on the approval of the study protocol must also be provided in the manuscript.

Field-specific reporting

Please select the one below that is the best fit for your research. If you are not sure, read the appropriate sections before making your selection.

☒ Life sciences ☐ Behavioural & social sciences ☐ Ecological, evolutionary & environmental sciences

For a reference copy of the document with all sections, see nature.com/documents/nr-reporting-summary-flat.pdf

Life sciences study design

All studies must disclose on these points even when the disclosure is negative.

Sample size	No statistical methods were used to predetermine the sample size. Sample sizes were determined based on published studies by our group, availability of the embryos/larvae and feasibility required to confirm obtained results. Overall, at least 5 samples per group were obtained for each statistical comparison.
Data exclusions	In CRISPR a/i experiments, samples with a single CM labeled were discarded as outliers. When obtaining data from whole-mount stained samples, hearts with severe distortion from the staining process were discarded to ensure accurate measurements. In strain analysis, samples with severe motion artifacts post-synchronization were discarded. During experiments, embryos that died or showed severe cardiac dysfunction before reaching end points were discarded. No other exclusion was carried out.
Replication	All experiments in this study were repeated independently at least twice with similar results (ISO treatment, ISO+ErbB2 inhibitor treatment,

	ISO+myosin inhibitor treatment, microbead insertion, TGF-b inhibition, and ISO+CRISPR activation/interference).
Randomization	Embryos/larvae were randomly selected and distributed into experimental groups from large pool of spawns.
Blinding	During imaging analysis, data were distributed to more than one investigators who did not perform the experiment, and the experimental conditions were concealed from them.

Reporting for specific materials, systems and methods

We require information from authors about some types of materials, experimental systems and methods used in many studies. Here, indicate whether each material, system or method listed is relevant to your study. If you are not sure if a list item applies to your research, read the appropriate section before selecting a response.

Materials & experimental systems

n/a	Involved in the study
☐	☒ Antibodies
☒	☐ Eukaryotic cell lines
☒	☐ Palaeontology and archaeology
☐	☒ Animals and other organisms
☒	☐ Clinical data
☒	☐ Dual use research of concern
☒	☐ Plants

Methods

n/a	Involved in the study
☒	☐ ChIP-seq
☒	☐ Flow cytometry
☒	☐ MRI-based neuroimaging

Antibodies

Antibodies used	We used anti-GFP (GeneTex GTX113617, 1:100), anti-mCherry (Invitrogen M11217, 1:100), and anti-MF20 (Invitrogen 14-6503-82, 1:500) primary antibodies. For secondary antibodies (1:500), we used Alexa Fluor 488/594/647 antibodies (Invitrogen A-11008, A-11007, A21235). A complete list of antibodies can be found in Supplementary Table 1.
Validation	Anti-GFP: GeneTex validated this antibody with various tests in line with guidelines described by the International Working Group on Antibody Validation (IWGAV).<br>Anti-mCherry: Invitrogen validated this antibody with various analysis including ICC, IHC, western blot, IP, and flow cytometry.<br>Anti-MF20: Invitrogen validated this antibody with various analysis including ICC, western blot, relative expression, and cell treatment.<br>The secondary antibodies are validated by Invitrogen via various ICC/IF tests.

Animals and other research organisms

Policy information about studies involving animals; ARRIVE guidelines recommended for reporting animal research, and Sex and Gender in Research

Laboratory animals	The study used zebrafish (Danio rerio) embryos (48 hpf, 56 hpf) and larvae (72 hpf, 96 hpf, 120 hpf, 6 dpf and 14 dpf). The following strains were used: Tg(myl7:mCherry), Tg(flk:mCherry), Tg(snai1b:EGFP)zd1100Tg, Tg(myl7:mCherry-zCdt1), Tg(TP1:EGFP)um14Tg, and Tg(myl7:mKate-CAAX).
Wild animals	No wild animals were used.
Reporting on sex	Our study focuses on time points prior to sex differentiation in zebrafish (~45 dpf).
Field-collected samples	No field samples were collected.
Ethics oversight	All experiments with zebrafish were performed in compliance with and with the approval of a UCLA Institutional Animal Care and Use Committee protocol (ID: ARC-2015-055).

Note that full information on the approval of the study protocol must also be provided in the manuscript.

Plants

Seed stocks	*Report on the source of all seed stocks or other plant material used. If applicable, state the seed stock centre and catalogue number. If plant specimens were collected from the field, describe the collection location, date and sampling procedures.*
Novel plant genotypes	*Describe the methods by which all novel plant genotypes were produced. This includes those generated by transgenic approaches, gene editing, chemical/radiation-based mutagenesis and hybridization. For transgenic lines, describe the transformation method, the number of independent lines analyzed and the generation upon which experiments were performed. For gene-edited lines, describe the editor used, the endogenous sequence targeted for editing, the targeting guide RNA sequence (if applicable) and how the editor was applied.*
Authentication	*Describe any authentication procedures for each seed stock used or novel genotype generated. Describe any experiments used to assess the effect of a mutation and, where applicable, how potential secondary effects (e.g. second site T-DNA insertions, mosiacism, off-target gene editing) were examined.*
